# Evaluation of Microflow Digital Imaging Particle Analysis for Sub-Visible Particles Formulated with an Opaque Vaccine Adjuvant

**DOI:** 10.1371/journal.pone.0150229

**Published:** 2016-02-29

**Authors:** Grant E. Frahm, Alex W. T. Pochopsky, Tessa M. Clarke, Michael J. W. Johnston

**Affiliations:** 1 Biologics and Genetic Therapies Directorate, Health Canada, Ottawa, Ontario, Canada; 2 University of Ottawa, Department of Biochemistry, Ottawa, Ontario, Canada; 3 University of Ottawa, Department of Mechanical Engineering, Ottawa, Ontario, Canada; Pennsylvania State University, UNITED STATES

## Abstract

Microflow digital imaging (MDI) has become a widely accepted method for assessing sub-visible particles in pharmaceutical formulations however, to date; no data have been presented on the utility of this methodology when formulations include opaque vaccine adjuvants. This study evaluates the ability of MDI to assess sub-visible particles under these conditions. A Fluid Imaging Technologies Inc. FlowCAM^®^ instrument was used to assess a number of sub-visible particle types in solution with increasing concentrations of AddaVax^™^, a nanoscale squalene-based adjuvant. With the objective (10X) used and the limitations of the sensor resolution, the instrument was incapable of distinguishing between sub-visible particles and AddaVax^™^ droplets at particle sizes less than 5 μm. The instrument was capable of imaging all particle types assessed (polystyrene beads, borosilicate glass, cellulose, polyethylene protein aggregate mimics, and lysozyme protein aggregates) at sizes greater than 5 μm in concentrations of AddaVax^™^ up to 50% (vol:vol). Reduced edge gradients and a decrease in measured particle sizes were noted as adjuvant concentrations increased. No significant changes in particle counts were observed for polystyrene particle standards and lysozyme protein aggregates, however significant reductions in particle counts were observed for borosilicate (80% of original) and cellulose (92% of original) particles. This reduction in particle counts may be due to the opaque adjuvant masking translucent particles present in borosilicate and cellulose samples. Although the results suggest that the utility of MDI for assessing sub-visible particles in high concentrations of adjuvant may be highly dependent on particle morphology, we believe that further investigation of this methodology to assess sub-visible particles in challenging formulations is warranted.

## Introduction

The presence of drug aggregates and sub-visible particles in therapeutic protein products has increasingly become a field of concern for both the pharmaceutical industry and regulatory agencies [1,2]. Aggregates in the micron range have been implicated in adverse reactions and/or reduction in efficacy of therapeutic products [1–6]. A number of factors can lead to the generation of protein aggregates. These include mechanical agitation (leading to exposure to hydrophobic air/water interfaces), chemical alteration, and/or temperature extremes [7]. Protein aggregates can also be generated through protein nucleation around nano/micro-scale contaminants in the product such as silica particles shed from containers, fibres shed from filters, or metal particles shed from production equipment [8,9].

Many techniques have been developed or adapted for quantifying and characterizing particles ranging in size from a few dozen nanometers (nm) (such as sub-visible aggregates) to tens of microns. Traditionally, dynamic light scattering has been used to size sub-micron (<1000 nm) particles (such as monomers, dimers and smaller oligomers), but cannot provide absolute particle counts and provides no information on particle morphology [10]. For larger particles, light obscuration (LO) or membrane microscopy were, until recently, considered the standard methods for particle counting [11]. However, these two techniques underestimate size and quantities for small transparent particles and cannot distinguish between particle populations [11]. These deficits lead to the development of microflow digital imaging (MDI) particle analysis, whereby particles are digitally imaged as they move through a flow cell. MDI is carried out using instruments such as the FlowCAM^®^ VS (Fluid Imaging Technologies, Scarborough, ME, USA) or MFI^™^ 5000 Series (Protein Simple, San Jose, CA, USA). It allows for the assessment of sub-visible aggregates in the range of 2 to 80 μm [12] with higher sensitivity for transparent particles and can differentiate subpopulations based on particle size, morphology, and optical density. Previous studies have compared LO methodology to MDI methodology for the characterization of protein particles and found that MDI was more sensitive for characterization of protein aggregates [6]. Earlier studies have utilized MDI to assess various materials, such as opalescent monoclonal antibody formulations [12] and recombinant *Mycobacterium tuberculosis* antigens adsorbed to liposomes [13]. These studies have shown that turbidity values of approximately 30–50 Formazin Nephelometric Units (FNU) had little effect on MDI techniques [14]. However, to the best of our knowledge, no studies have utilized micro flow imaging techniques for the assessment of protein aggregates when samples are formulated with an opaque vaccine adjuvant. This study evaluates the utility of MDI particle analysis, specifically using the FlowCAM^®^ VS instrument, to assess a variety of sub-visible particles in the presence of a squalene-based vaccine adjuvant. The results of this investigation will be of interest to those assessing sub-visible particles, especially those that are opaque, in vaccines where a squalene adjuvant is added prior to distribution [15] or for vaccines in which the adjuvant is mixed with the vaccine at the time of administration [16] and sub-particle analysis is required after formulation in the clinic.

## Materials and Methods

### Materials

All reagents were supplied by Sigma-Aldrich Co. (St. Louis, MO, USA), unless otherwise specified. AddaVax^™^, a squalene-based oil-in-water nano-emulsion similar to MF59^®^ and shown to be effective as an influenza vaccine adjuvant [17], was obtained from Invivogen (San Diego, Ca, USA). NIST traceable polystyrene COUNT-CAL^™^ particle size standards (5, 10, 20 and 50 μm, referred to hereafter as PS beads) were purchased from Thermo Scientific (Waltham, Massachusetts, USA).

### Particle Sample Preparation

Proteins were buffer exchanged into 10 mM citrate pH 6.5 using EMD Millipore Amicon Ultra 3K 0.5 mL centrifuge tubes which were spun at 10,000 g in a Thermo Scientific Sorvall ST 40R centrifuge. The concentration of this protein was then determined using a BCA assay kit. Generation of protein aggregates was accomplished by rapidly heating lysozyme to 70°C for 15 seconds followed by stirring at 37°C for 168 hours (one week).

Ultra high molecular weight polyethylene (UHMWPE) (Lee Valley Tools Ltd., Ogdensburg, NY, USA) was used to generate stable particles that mimic protein aggregates. Particles were generated by wet sanding (citrate buffer + 0.5% TWEEN 20) solid stock UHMWPE with 600 grit wet/dry sand paper similar to methods described previously [18]. The resulting slurry was washed through a 10 μm filter (Clear Edge Filtration Canada, Guelph, Ontario, Canada). Due to the potential for UHMWPE particle/particle adhesion, and/or UHMWPE particle adhesion to containers, particle mimics were formulated with TWEEN 20. Borosilicate glass particles were prepared by milling borosilicate glass Pasteur pipettes with a ceramic mortar and pestle. As with the UHMWPE protein mimics, the resulting particles were washed and filtered through a 10 μm filter with the retentate further washed with citrate buffer through 50 μm filters.

### Assessment of adjuvant turbidity and size

Absorbance of polymer bead turbidity standards was assessed at 350nm on a Biochrom^™^ Ultrospec 3100 Pro (Fisher Scientific Company, Ottawa, Ontario, Canada) at room temperature, similar to previously published studies [19,20], to produce a standard curve. AddaVax^™^ formulations were diluted as appropriate to ensure their absorbance fell on the standard curve and read at 350 nm. Particle sizing of AddaVax^™^ samples was performed with a NanoSight NS300 (Malvern Instruments, Malvern UK) at room temperature according to the manufacturer’s instructions.

### Flow Imaging Microscopy

A Fluid Imaging Technologies Inc. (Scarborough, Maine, USA) FlowCAM^®^ model Benchtop B3 Series (renamed VS series) fitted with a 10x objective lens was used to visualize each sample run. FC100 (100 x 2000 μm) flow cells were manually focused using 50 μm PS beads in citrate buffer and used for no more than 10 runs (analysis parameters included: 0.85 mL sample analysed per run, 0.90 mL sample loaded, Distance to Nearest Neighbour = 3 μm, AutoImage Frame Rate = 22 fps and Flash Duration = 22.50 μs). Focus was considered acceptable when the mean edge gradient was above 150 gradients for 50 μm PS beads (gradient describes the intensity change from background to foreground, where high values indicate a more rapid transition from background to particle and therefore a more sharply focused edge). The instrument segmentation threshold values were set for each particle type (S1 Table).

Between each run, the flow cell was washed with 1.5 mL of a 5% Tween 20 solution, and then rinsed with 15 mL of water. PS particle standards, borosilicate glass particles, cellulose particles (Thermo Scientific, Waltham, Massachusetts, USA), protein aggregates, protein aggregate mimics and influenza vaccine samples were formulated without and with AddaVax^™^ at appropriate ratios (vol:vol) and analysed by MDI in a similar fashion. Samples without AddaVax^™^ were prepared with citrate buffer in place of the adjuvant. When assessing particle standards, data was filtered for appropriate sizes (for example, for 20 μm PS beads, only particles in the size range of 18 to 22 μm were counted). When assessing particle types, only particles larger than 5 μm were quantified. Particle duplicates or particle fragments were manually removed from the data prior to analysis even after optimizing segmentation threshold settings (all studies here were conducted by a single operator to minimize operator to operator variability). PS particle diameters were determined through equivalent spherical diameter calculations and all non-PS particles types through area-based diameter calculations where the Visual Basic software calculates a diameter of a circle with an equivalent area of the sample particle.

### Statistical Analysis, Particle Sizing and Counting Precision

Each result is presented as the mean ± standard deviation of at least 3 separate experiments for PS particle standards, borosilicate, cellulose and UHMWPE protein aggregate mimics and three separate experiments of three technical replicates for lysozyme protein aggregates. The Student’s t-test was utilized to assess significant differences in mean particle counts, particle size and particle edge gradient for all particle types in varying concentration of AddaVax^™^ compared to AddaVax^™^-free formulations. Analyses were carried out with SigmaPlot 12.5 software (Systat Software, Inc., San Jose, CA, USA) and significance was designated as p < 0.05.

The percent sensor resolution (the smallest change that the sensor can detect for the quantity it is measuring) was determined by calculating the coefficient of variance (*CV*_*i*_) according to previously published methodology:

$$CV_{i}=\frac{\sqrt{sd^{2}obs+sd^{2}p}}{D_{p}}$$

where *sd*^*2*^*obs* is the observed particle variance, *sd*^*2*^_p_ is the manufacturer reported particle size variance and *D*_*p*_ is the supplied particle diameter. Instruments with coefficient of variance for particle diameters of less than 10% (for well-defined particles) are considered acceptable for characterization of sub-visible particulate matter in injections and ophthalmic solutions according to the USP [14,21]. The coefficient of variance for particle counting was calculated by dividing the standard deviation for a particle size group by the mean number of particles counted for that size range.

## Results

### Characterization of AddaVax^™^ Adjuvant

**Fig 1:**
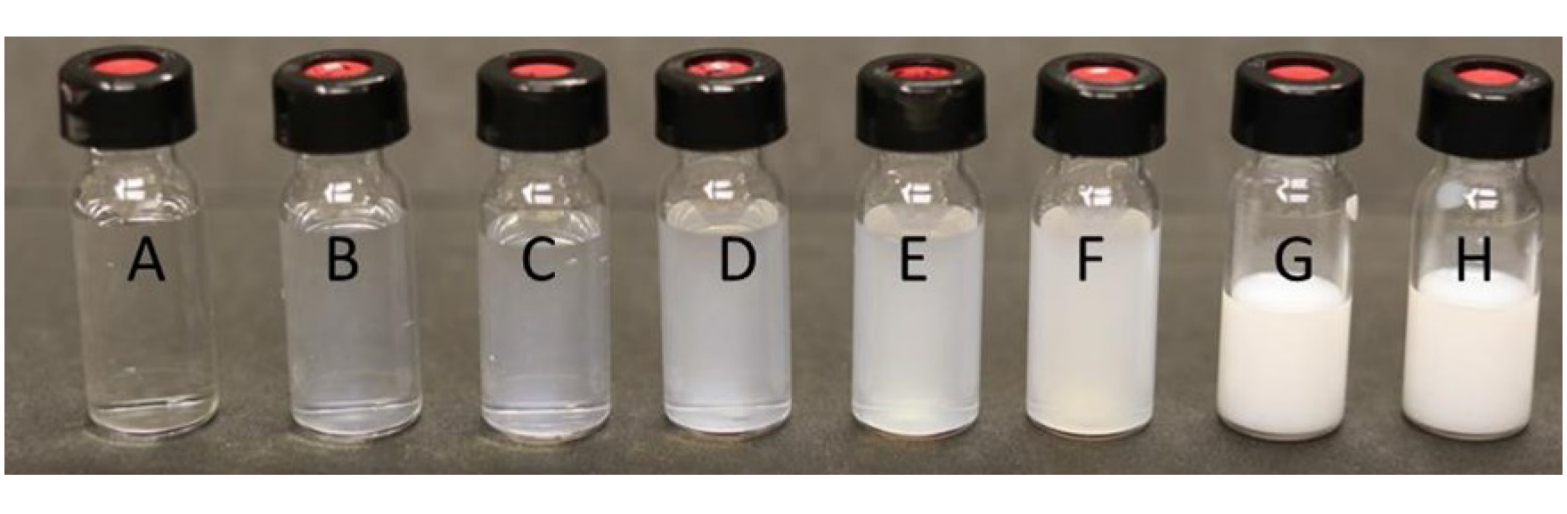
Turbidity standards. Vials A through F are 0, 50, 100, 250, 500 and 1000 NTUs respectively. Vial G is 50% unfiltered AddaVax^™^ and vial H is 50% 0.22 μm filtered AddaVax^™^, both in citrate buffer.

**Fig 2:**
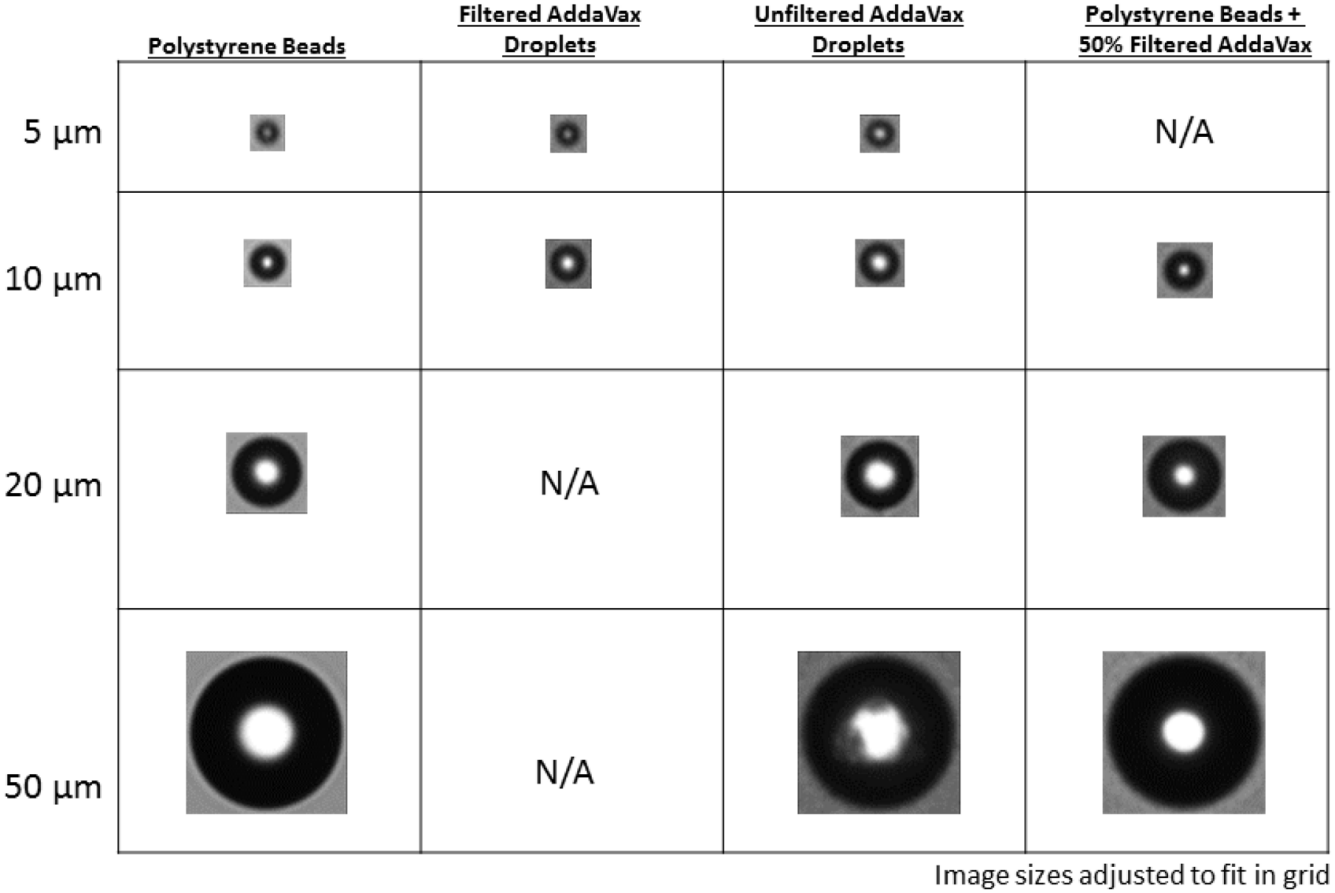
Representative images (sharpest selected) of COUNT-CAL particle standards and large droplets of AddaVax^™^.

Vaccines adjuvanted with squalene-based adjuvants are typically formulated in a 1:1 ratio of antigen to adjuvant (vol:vol) and result in an opaque mixture (Fig 1) with a turbidity measure of approximately 16000 Nephelometric Turbidity Units (NTUs). When AddaVax^™^ was diluted 1:1 with citrate buffer; microflow digital imaging (MDI) showed the presence of a large number of spherical particles which have similar size and morphology to 20 μm PS beads (Fig 2). A limited number of these particles were also similar in size to the 50 μm PS beads. Filtering AddaVax^™^ through a 0.22 μm syringe filter removed the majority of these particles larger than 10 μm. Further characterization of filtered AddaVax^™^ with nanoparticle tracking analysis showed that the filtered adjuvant had an average particle size of 116.1 +/- 24.1nm.

### Assessment of PS Beads in AddaVax^™^

MDI particle analysis with the FlowCAM^®^ VS instrument of 20 and 50 μm PS beads in increasing concentrations of AddaVax^™^ showed that the adjuvant had no significant impact on the accuracy or precision of the instrument when measuring particle size or number. Analysis of 20 μm PS beads (manufacturer’s approximate concentration = 3000 ± 300 particles/mL) showed that there was no effect on particle counts as filtered AddaVax^™^ concentrations increased (Fig 2, Table 1). The instrument was also capable of accurately determining particle size, with no significant difference to the NIST traceable mean diameter of 19.99 +/- 0.28 μm at all adjuvant concentrations (Table 1). Increasing PS bead size to 50 μm allowed for accurate particle counts in both unfiltered and filtered AddaVax^™^ (manufacturer’s approximate concentration of 3000 +/-300 particles/ml) (Table 2). Increasing adjuvant concentrations also had no significant effect on measured particle size (NIST traceable size of 50.2 +/- .05μm).

**Table 1:** Assessment of 20 μm beads in filtered AddaVax^™^.

AddaVax^™^ concentration	Particles/ml*	CV_i_ (particles/ml)	Particle size (μm)*	CV_i_ (particle size)	Mean Edge Gradient*
0%	2829.3 +/- 23.10	0.01	19.38 +/- 0.67	0.03	146.0 +/- 9.8
10%	2712.0 +/- 157.8	0.06	19.43 +/- 0.72	0.03	123.3 +/- 4.1**
25%	2816.7 +/- 122.3	0.04	19.13 +/- 0.78	0.04	106.1 +/- 30.9
50%	2783.3 +/- 182.6	0.07	18.70 +/- 0.79	0.04	99.1 +/- 20.6**

* Value is the mean of 3 separate experiments +/- standard deviation

** Statistically relevant difference in comparison to AddaVax^™^-free samples

**Table 2:** Assessment of 50 μm beads in unfiltered and filtered AddaVax^™^.

Unfiltered AddaVax^™^ Concentration	Particles/ml*	CV_i_ (Particle/ml)	Particle size (μm)*	CV_i_ (Size)	Mean Edge Gradient*
0%	3183.5 +/- 348.4	0.11	49.37 +/- 2.24	0.04	174.1 +/- 1.7
10%	3232.8 +/- 430.0	0.13	49.60 +/- 2.24	0.04	136.8 +/- 47.0
25%	3140.7 +/- 438.1	0.14	49.51 +/- 2.97	0.06	116.3 +/- 33.6
50%	3206.0 +/- 261.5	0.08	49.02 +- 2.46	0.05	106.6 +/- 20.5**
Filtered AddaVax^™^ Concentration	Particles/ml*	CV_i_ (Particle/ml)	Particle size (μm)*	CV_i_ (Size)	Mean Edge Gradient*
0%	3046.7 +/- 139.4	0.05	49.55 +/- 2.00	0.04	173.6 +/- 0.5
10%	3092.7 +/- 405.1	0.13	49.59 +/- 2.11	0.04	148.8 +/- 27.0
25%	2794.7 +/- 238.7	0.09	49.35 +- 2.57	0.05	140.8 +/- 24.2
50%	3012.7 +/- 234.0	0.08	48.70 +- 2.79	0.05	126.4 +/- 6.9**

* Value is the mean of 3 separate experiments +/- standard deviation

** Statistically relevant difference in comparison to AddaVax^™^-free samples

A non-significant pattern toward smaller particle size assignments was noted for PS beads as the concentration of adjuvant increased. Correlating with this pattern was a significant decrease in observed edge gradients at adjuvant concentrations of 50%. This loss of optical contrast and blurring at the edges of the PS particle was demonstrated most plainly for the 50 μm PS beads in Fig 2, and could possibly result in the instrument software assigning a smaller particle size with greater variability. Supporting this interpretation are previous studies showing that reduced differences in refractive indices between the particle and the solution lead to reductions in measured particle size and particle count [14,22].

### Assessment of Cellulose and Borosilicate in AddaVax^™^ Adjuvant

**Fig 3:**
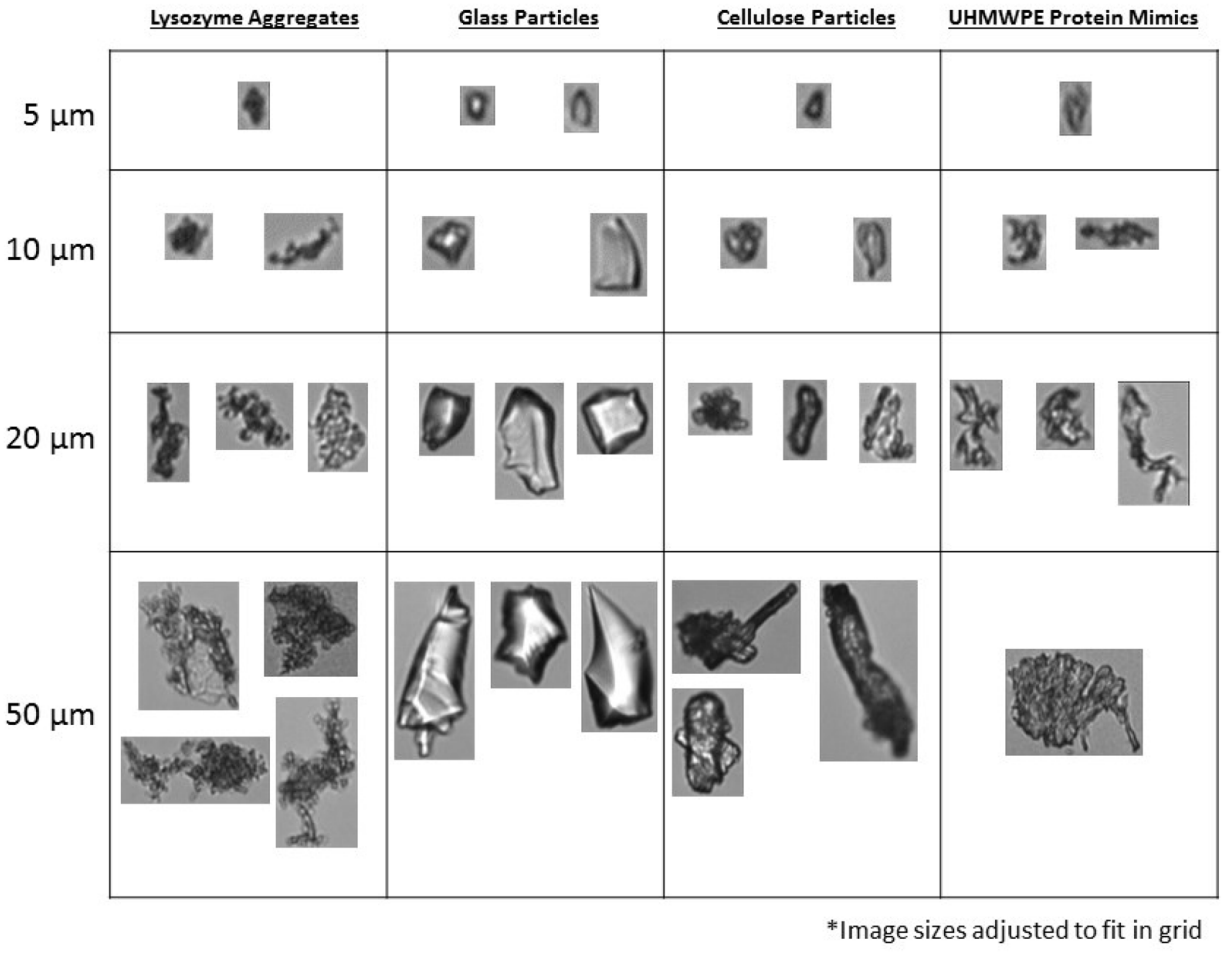
Representative images obtained from FlowCAM^®^ VS assessment of particles used in this study at various size ranges. All images were taken in the absence of AddaVax^™^.

**Fig 4:**
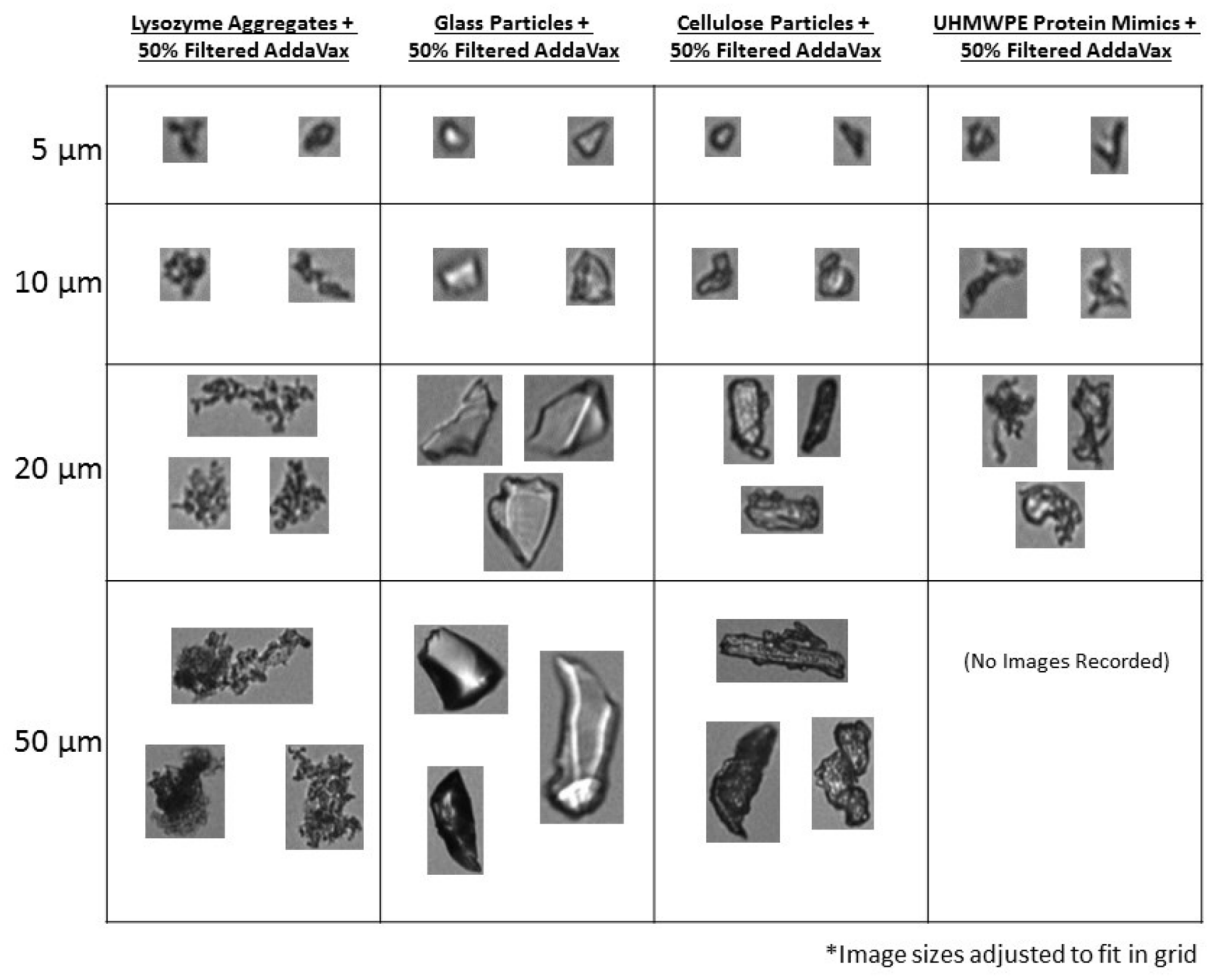
Representative images of particles used in this study at various size ranges obtained from FlowCAM^®^ VS assessment of particles in 50% AddaVax^™^.

**Fig 5:**
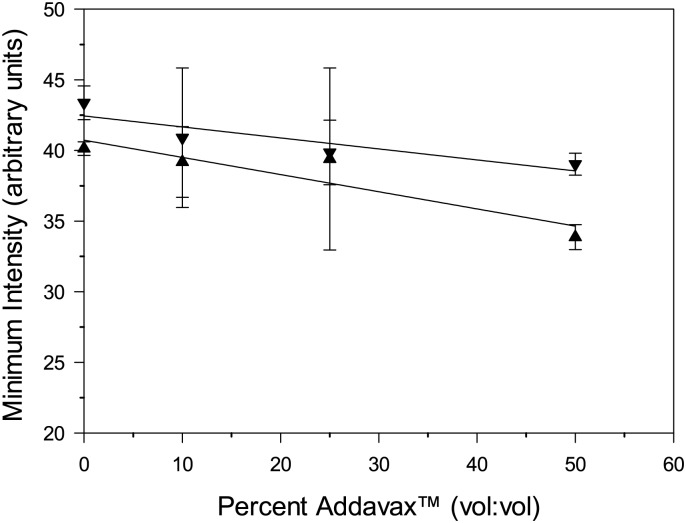
Mean minimal intensity for borosilicate (▲) and cellulose (▼) in increasing concentrations of AddaVax^™^. Data points represent mean values of three separate experiments and error bars represent the standard deviation.

Although the FlowCAM^®^ VS could readily quantify and characterize PS beads in high concentrations of adjuvant, these particles are not representative of typical sub-visible particles of interest, such as those composed of borosilicate or cellulose. Previous studies have shown that borosilicate particles in pharmaceutical formulations may originate from vials and fill containers, and cellulose particles from cellulose-based filters [23,24]. Representative images of cellulose and borosilicate particles taken from FlowCAM^®^ VS analysis are shown without AddaVax^™^ in Fig 3 and formulated with 50% AddaVax^™^ in Fig 4. The borosilicate particles had a mean particle size of 12.8 μm and cellulose particles appeared distinctly different, with a mean particle size of 16.8 μm. Assessing cellulose and borosilicate particles in increasing concentrations of AddaVax^™^ showed a significant reduction in measured particle concentrations at 50% AddaVax^™^, with 94% and 80% of the original particle concentration measured for cellulose and borosilicate particles, respectively (Table 3). A significant reduction in measured edge gradients for borosilicate particles was observed in the 50% AddaVax^™^ formulations (Table 3). The mean minimal particle intensity is a measure of the transparency of the sample [22], or conversely, of the degree to which particles are masked by the surrounding solution. It is calculated as the average grayscale value of pixels on a scale of 0–255, with 0 indicating maximum transparency and 255 indicating maximum particle masking. This value is typically lower for borosilicate particles than cellulose particles, as shown in Fig 5.

**Table 3:** Assessment of ≥5 μm cellulose and borosilicate particles in increasing concentrations of filtered AddaVax^™^.

Filtered AddaVax^™^ Concentration	Cellulose particles/ml*	Mean particle size (μm)*	Mean Edge Gradient*
0%	63609 +/- 1334	12.8 +/- 0.4	89.2 +/- 7.6
10%	63117 +/- 992	12.4 +/- 0.0	93.8 +- 2.4
25%	60765 +/- 887**	12.3 +/- 0.3	87.5 +/- 7.6
50%	59588 +/- 2333**	12.3 +/- 0.1	87.0 +/- 2.8
Filtered AddaVax^™^ Concentration	Borosilicate particles/ml*	Mean particle size (μm)*	Mean Edge Gradient*
0%	11281 +/- 274	16.8 +/- 0.8	99.7 +/- 4.3
10%	10348 +/- 760	15.5 +/- 0.4	101.6 +/- 1.0
25%	9240 +/- 503**	15.6 +/- 0.6	96.0 +/- 3.1
50%	9077 +/- 504**	15.5 +/- 0.4	87.1 +/- 3.4**

* Value is the mean of 3 separate experiments +/- standard deviation

** Statistically relevant difference in comparison to AddaVax^™^-free samples

### Assessment of Protein Aggregate Mimics and Protein Aggregates in the Presence of Squalene Based AddaVax^™^

To further examine the utility of the FlowCAM^®^ VS instrument in characterizing and quantifying sub-visible particles in highly opaque adjuvanted solutions, UHMWPE protein aggregate mimics were produced (Fig 3). These protein aggregate mimics were morphologically similar to previous examples of polymer-based protein aggregate mimics generated with similar methodology [18,25] and dramatically different from either the PS beads, cellulose or borosilicate particles. When these mimics were formulated with increasing AddaVax^™^ concentrations, the FlowCAM^®^ VS could readily identify them (Fig 4) and showed an average particle size of 8.1 μm (Table 4). Particle counting of the protein aggregate mimics showed no statistically relevant change in particle counts as AddaVax^™^ concentrations increased, but a trend was noted for reduced particle concentrations at the higher AddaVax^™^ concentrations (Table 4). A statistically relevant reduction in mean particle size and edge gradients was also observed (Table 4) for the samples with 50% adjuvant concentration. Similar to PS beads, the reduced edge gradient could be due to edge blurring by the adjuvant leading to reduced measured particle sizes, although this was not as clearly evident as the edge blurring with the 50 μm PS beads. A higher power objective may provide more information in this area.

**Table 4:** Assessment of ≥5 μm UHMWPE protein aggregate mimics in increasing concentrations of AddaVax^™^.

Filtered AddaVax^™^ Concentration (vol/vol)	Protein aggregate mimic particles/ml*	Mean Particle Size (μm)*	Mean Edge Gradient*
0%	26804 +/- 1174	8.1 +/- 0.1	100.0 +/- 4.5
10%	28433 +/- 1042	8.1 +/-0.1	99.4 +/- 2.4
25%	25313 +/- 815	8.1 +/- 0.1	95.6 +/-1.6
50%	24732 +/- 1249	7.8 +/- 0.1**	88.7 +/- 2.0**

* Value is the mean of 3 separate experiments +/- standard deviation

** Statistically relevant difference in comparison to AddaVax^™^ free sample

A major objective in measuring sub-visible particles in protein drug formulations is the assessment of protein aggregates due to their influence on efficacy and safety [23,24]. Although synthetic protein aggregate mimics can resemble some types of protein aggregates, protein aggregates observed in previous studies can have a variety of morphologies and opacities [11]. Protein aggregate morphology can be influenced by the specific protein being aggregated, the age of the sample, the formulation of the sample and the method of aggregation induction. For instance, chemical denaturation of a monoclonal antibody yielded opaque particles whereas agitation-induced aggregations of the same mAb generated transparent aggregates (10).

We examined sub-visible protein aggregates from five proteins (lysozyme, BSA, HSA from human plasma, recombinant HSA from Rice, and recombinant transferrin from rice, S1 Fig), all of which showed similar morphology. These particles also had similar morphologies to proteinaceous aggregates of chemically denatured monoclonal antibodies [12]. Of these, lysozyme generated the most consistent particle concentrations and was used for all further studies (Figs 3 and 4). It has been suggested that the use of generated protein particles is not ideal due to the potential for changes in consistency of particle generation and the possible instability of the aggregates [14]. This was not the case here, as lysozyme aggregates formulated with 50% filtered AddaVax^™^ (vol:vol) could be readily identified and characterized (Figs 3 and 4). At particle sizes less than 10 μm, due to the resolution of the instrument’s optics, both the lysozyme aggregates and the UHMWPE protein aggregate mimics were similar in appearance. However, at large particle sizes, distinct differences could be seen with UHMWPE protein mimics resembling opaque sheets and lysozyme aggregates appearing much more granular.

No statistically relevant reductions in particle size and edge gradient were noted and virtually no differences in particle counts were observed between samples with and without the adjuvant, suggesting that these aggregates are highly stable (Table 5). This data suggests that MDI particle analysis with the FlowCAM VS instrument is capable of assessing particle aggregates in highly opaque adjuvanted solutions. Ideally, certified standards should/would be made commercially available which mimic a wide variety of protein aggregate morphologies. Further studies with higher repetitions of such standards could be used to validate the results reported here.

**Table 5:** Assessment of ≥5 μm lysozyme aggregates in 50% AddaVax^™^.

W/O AddaVax^™^	Exp. 1	Exp. 2	Exp. 3	Exp. 4	Mean
Particle/ml	1226	1249	1249	1168	1223 +/- 38
Mean Particle Size (μm)	18.9	21.6	17.2	17.2	18.7 +/-2.1
Measured Edge Gradient	97.0	95.9	89.41	76.41	89.7+/-9.5
W AddaVax^™^ (50%, vol:vol)	Exp. 1	Exp. 2	Exp. 3	Exp. 4	Mean
Particle/ml	1133	1293	1214	1098	1185 +/- 87
Mean Particle Size (μm)	19.5	20.5	18.1	14.2	18.1 +/-2.7
Measured Edge Gradient	85.6	80.2	84.6	69.2	79.9 +/-7.5

### Assessment of Adjuvanted Influenza Vaccine with Microflow Digital Imaging

**Fig 6:**
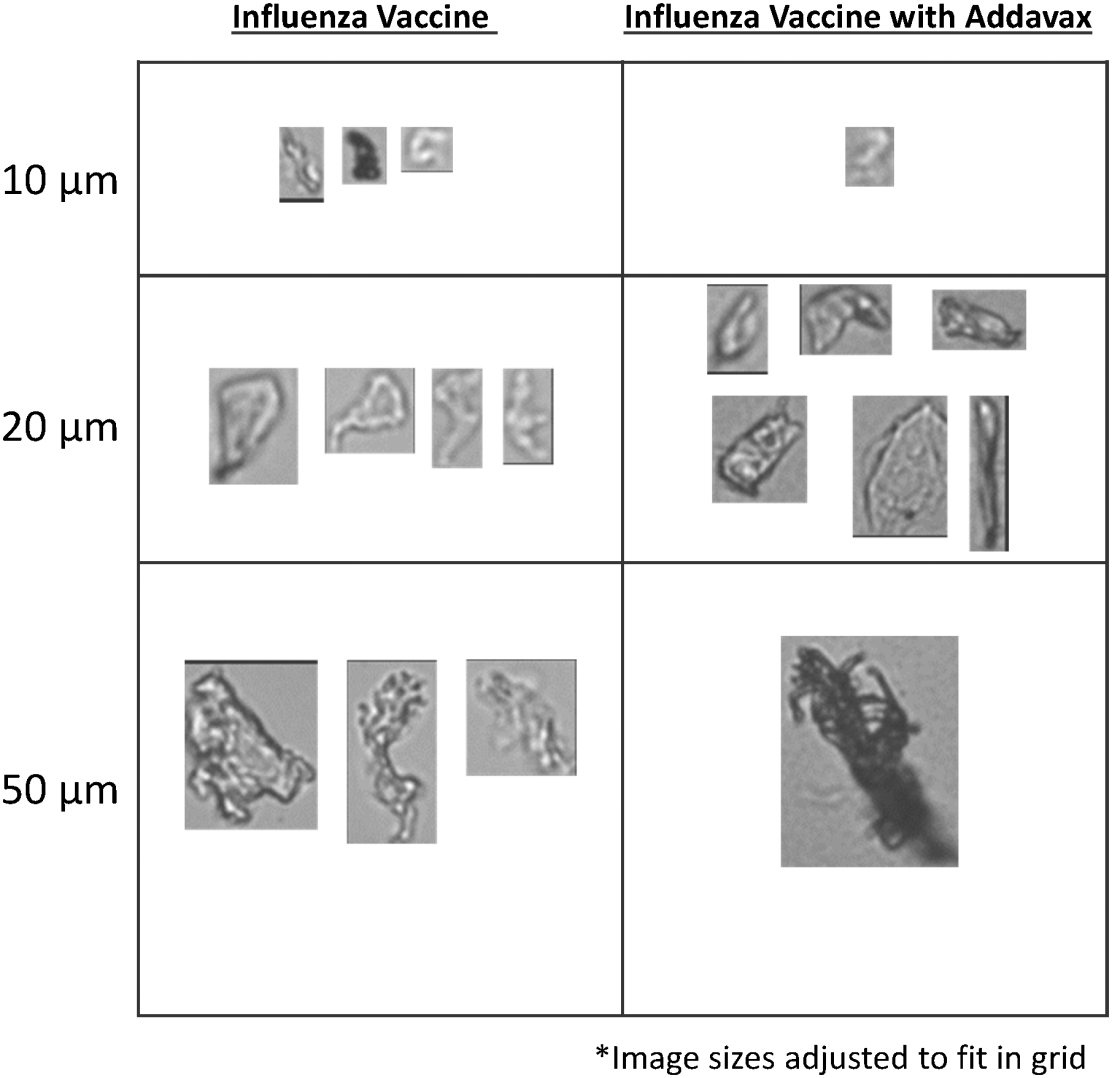
Representative images of particles at various size ranges obtained from FlowCAM^®^ VS assessment of influenza vaccine formulated without and with AddaVax^™^.

We assessed the presence of sub-visible particles in an influenza vaccine (undisclosed manufacture, expired) with and without the presence of 50% filtered AddaVax^™^ (vol:vol). Due to a limited supply of material only two repeats were performed, but a clear reduction in particle counts with no change in mean particle size can be observed when adjuvant is included in the formulation (Table 6). Examination of particle morphology (Fig 6) shows a lack of detection of light/transparent particles when the adjuvant is present, which suggests that like borosilicate particles, the adjuvant is masking particles with this morphology.

**Table 6:** Assessment of ≥5 μm sub-visible particles in an influenza vaccine when formulated with 50% AddaVax^™^.

Sample	Influenza Vaccine W/O Addavax Rep #1	Influenza Vaccine W/O Addavax Rep #2	Influenza Vaccine W Addavax Rep #1	Influenza Vaccine W Addavax Rep #2
Particle/ml	15002	14993	4647	5630
Mean Particle Size (μm)	6.3	6.3	6.4	6.8
Measured Edge Gradient	166.3	165.9	137.4	127.0

## Discussion

A correlation between the presence of sub-visible proteinaceous particles and loss of efficacy and/or immunogenicity for protein based therapeutics has been identified [3,26,27]. The development of MDI instrumentation has allowed for more sensitive assessment of the proteinaceous particles compared to existing techniques such as LO. Studies have shown that LO underestimates both the number and size for proteinaceous particles, is unable to differential sub-populations of particles, and is more highly affected by the refractive index of the solution compared to MDI [6,11,12,28].

Previous studies have investigated the utility of MDI in a variety of solution conditions including increased turbidity of up to 60 NTUs with little or no effect on particle characterization performance [14]. In this study we attempted to assess the utility of MDI under even more challenging solution conditions, specifically the presence of an opaque vaccine adjuvant. Although our data shows that MDI particle analysis with the FlowCAM VS instrument is capable of characterizing and quantifying a number of particle types in highly opaque adjuvanted solutions, a number of limitations need to be discussed.

The first is the loss of optical contrast and blurring at the edges of the PS particle as the concentration of adjuvant increase, as is demonstrated most plainly for the 50 μm PS beads in Fig 2. This could explain the slightly reduced particle size and increased measurement variability as Addavax concentration increased. Supporting this are previous studies showing that reduced differences in refractive indices between the particle and the solution lead to reductions in measured particle size and particle count [14,22,28].

Secondly, although the FlowCAM^®^ VS could readily quantify and characterize PS beads in high concentrations of adjuvant, these particles are not representative of typical sub-visible particles of interest, such as those composed of borosilicate, cellulose or protein. Our results show that although MDI could characterize and quantify these particles types in high concentrations of Addavax, a reduction in particle counts for borosilicate particles or particles in an influenza vaccine in 50% AddaVax^™^ was observed. The morphological differences between borosilicate particles and other particle types may offer an explanation for this. An examination of representative images of cellulose particles shows that the majority of these particles have dark opaque edges whereas the borosilicate particles can have lighter perimeters, most likely along sharp beveled edges (Fig 3). The increased opacity of the adjuvanted solutions may mask these translucent borosilicate particles to a greater degree than the cellulose, PS, proteinaceous particles or UHMWPE protein mimic particles, which are more opaque and/or have well-defined edges even at higher AddaVax^™^ concentrations. The decreased contrast between the particle and the solution background could be below the threshold required for the system to recognize a particle. This could result in particle fragmentation leading to smaller calculated mean particle sizes, or particles being ignored completely resulting in reduced particle counts [11]. The differences in counting efficiency between the transparent and opaque particles at high concentrations of adjuvant suggest the utility MDI may be dependent on particle morphology. In these conditions, MDI may only be capable of accurately quantifying sub-visible particles having high opacity (metallic particles, cellulose particles or proteinaceous particles). This is further demonstrated by the assessment of sub-visible particles in influenza vaccine samples when formulated with 50% AddaVax^™^. The majority of these particles were highly transparent resulting in over one half of them not being detected when the adjuvant is present at this concentration. Presumably these transparent particles are membrane debris from the manufacturing process and an orthogonal, non-optical technique such as resonant mass measurement [29] would be required for complete sub-visible particle characterization of these types of vaccines.

Finally, owing to the objective (10X) fitted to our instrument, a lower limit of 5 μm particle diameter was set for sub-visible particle counting; the resolution of the instrument at this magnification did not allow for differentiation between residual AddaVax^™^ particles and borosilicate or cellulose particles smaller than 5 μm. The use of a higher power objective (20X) could provide the increased resolution required to better differentiate between particle types in this size range, but would necessitate a narrower flow cell, preventing the assessment of larger aggregates. To overcome these limitations, the samples could be run twice with different objectives for each run providing data for a wider range of particle sizes.

## Conclusions

The goal of this study was to assess the capability and ascertain the limits of the FlowCAM^®^ VS instrument and MDI particle analysis to evaluate sub-visible protein aggregates when samples contain high concentrations of an opaque vaccine adjuvant. It should be noted that this study was not designed to validate the instrument or method under our assay conditions and these results should be viewed, similar to previous studies (12), as preliminary; useful to direct further research where a higher number of experimental repetitions would provide more powerful statistical analysis. Regardless of the caveats we have noted for the assessment of sub-visible particles in highly opaque solutions by MDI particles analysis, we believe that further investigation of this methodology to assess protein particles in challenging formulations is warranted. The availability of well characterized protein particle standards, such as those developed by NIST [18,25], will allow for a more specific determination of the extent to which particle morphology plays a role in the ability to accurately characterize particles in solutions with high opacity. We also believe that other vaccine formulations, such as liposome adjuvanted vaccines [30] or highly purified subunit vaccines adjuvanted with oil in water emulsions [15,31], should be investigated.

## Supporting Information

S1 Fig Representative images obtained from FlowCAM^®^ VS assessment of sub-visible particles from various proteins at various size ranges. All images were taken in the absence of AddaVax^™^.
(TIF)

S1 Table Parameters for FlowCAM measurements. (DOCX)
