# Supplementary figures and images for: Evaluation of Microflow Digital Imaging Particle Analysis for Sub-Visible Particles Formulated with an Opaque Vaccine Adjuvant
Source: PLoS One. 2016 Feb 29;11(2):e0150229. doi: 10.1371/journal.pone.0150229 (PMC4771808; doi:10.1371/journal.pone.0150229)

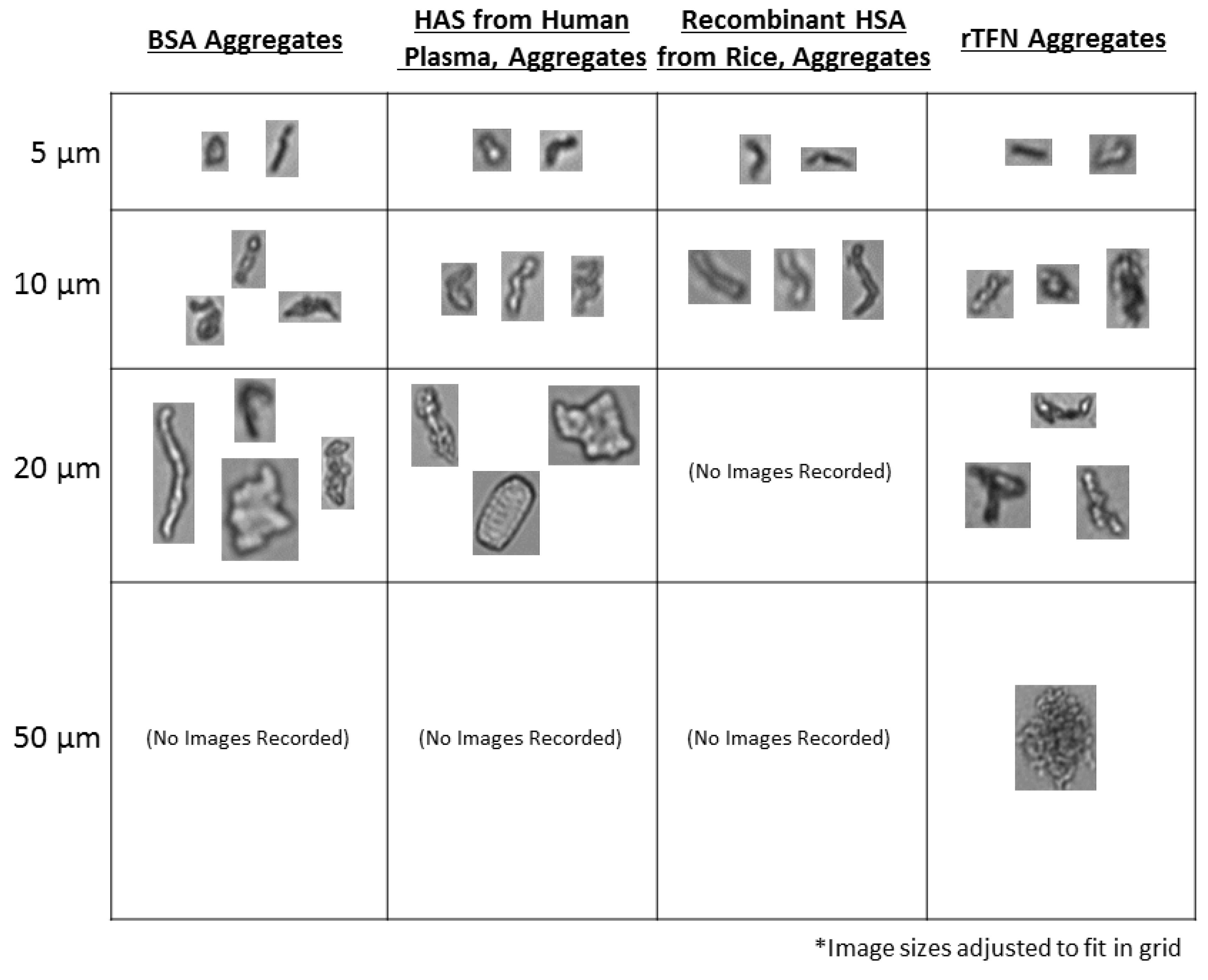

Supplement: S1 Fig — All images were taken in the absence of AddaVax™. (TIF) [file pone.0150229.s001.tif]
