# Supplementary material for: Evaluation of Microflow Digital Imaging Particle Analysis for Sub-Visible Particles Formulated with an Opaque Vaccine Adjuvant
Source: PLoS One. 2016 Feb 29;11(2):e0150229. doi: 10.1371/journal.pone.0150229 (PMC4771808; doi:10.1371/journal.pone.0150229)
Supplement: S1 Table — (DOCX) [file pone.0150229.s002.docx]

| Sample | Threshold Settings | Size Calculation |
| --- | --- | --- |
| PS Beads | 50 Dark/0 Light | Equivalent Spherical Diameter |
| Borosilicate particles | 20 Dark/0 Light | Area Based Diameter (ABD) |
| Cellulose particles | 20 Dark/0 Light | ABD |
| Protein Aggregates/UHMWPE protein aggregate mimics | 30 Dark/30 Light | ABD |
